# ModelBricks—modules for reproducible modeling improving model annotation and provenance

**DOI:** 10.1038/s41540-019-0114-3

**Published:** 2019-10-08

**Authors:** Ann E. Cowan, Pedro Mendes, Michael L. Blinov

**Affiliations:** 1 Center for Cell Analysis and Modeling, UConn Health, Farmington, CT USA; 2 Department of Molecular Biology and Biophysics, UConn Health, Farmington, CT USA; 3 Center for Quantitative Medicine, UConn Health, Farmington, CT USA; 4 Department of Cell Biology, UConn Health, Farmington, CT USA; 5 Department of Genetics and Genome Sciences, UConn Health, Farmington, CT USA

**Keywords:** Modularity, Software, Complexity, Computer modelling

## Abstract

Most computational models in biology are built and intended for “single-use”; the lack of appropriate annotation creates models where the assumptions are unknown, and model elements are not uniquely identified. Simply recreating a simulation result from a publication can be daunting; expanding models to new and more complex situations is a herculean task. As a result, new models are almost always created anew, repeating literature searches for kinetic parameters, initial conditions and modeling specifics. It is akin to building a brick house starting with a pile of clay. Here we discuss a concept for building annotated, reusable models, by starting with small well-annotated modules we call ModelBricks. Curated ModelBricks, accessible through an open database, could be used to construct new models that will inherit ModelBricks annotations and thus be easier to understand and reuse. Key features of ModelBricks include reliance on a commonly used standard language (SBML), rule-based specification describing species as a collection of uniquely identifiable molecules, association with model specific numerical parameters, and more common annotations. Physical bricks can vary substantively; likewise, to be useful the structure of ModelBricks must be highly flexible—it should encapsulate mechanisms from single reactions to multiple reactions in a complex process. Ultimately, a modeler would be able to construct large models by using multiple ModelBricks, preserving annotations and provenance of model elements, resulting in a highly annotated model. We envision the library of ModelBricks to rapidly grow from community contributions. Persistent citable references will incentivize model creators to contribute new ModelBricks.

## Introduction

Computational models are increasingly used to interpret experimental data and to integrate various sources of information. In their most sophisticated (and useful) form, models include quantitative parameter values and mathematical expressions that are precise expressions of prior knowledge and assumptions and allow them to be executable; their simulation predicts system behavior and generates new hypotheses and new insights into biological phenomena. Quantitative, executable models are thus rich and highly structured data objects integrating information from diverse sources and there are databases and formal identifiers for most components of a biological model.^1–11^ Ultimately, this rich annotation becomes orders of magnitude more useful when it becomes easily transferred among different modeling tools and strategies. Standards have been developed that enable mathematical model exchange,^12,13^ for model visualization,^14^ for pathways description,^15^ and for simulation specifications.^16^ Many software tools are compliant with the Minimal Information Required in the Annotation of Models,^17^ and thus provide ways to store annotations and provide some assistance to the model builder in annotating their model.

The large amount of modeling data that already exists often cannot be reused because the data lacks proper annotation. An exception is the BioModels Database,^18^ which houses extensively annotated and curated models; models are stored in SBML format and curated to ensure rich structured annotation with references to external data sources. BioModels Database includes more than 700 curated models and, in addition, more than 7000 non-curated models, all freely available under the Creative Commons CC0 license.^19^ A database associated with the Virtual Cell (VCell) modeling environment,^20^ VCell Database (VCellDB), also stores models and simulation results that can be shared, reused, updated, and made available to the scientific community. Close to 70,000 models are now stored in VCellDB with almost 1000 models openly available to the public. The Aukland Physiome Repository houses the CellML database^21^ with over 630 models in various states of curation. ModelDB hosted at the SenseLab at Yale University^22^ houses over 1000 computational neuroscience models that are largely created with the popular Neuron software. Combined, these model databases represent rich sources of information that could be leveraged to simplify model building by the community. However, while BioModels uses a well-structured and formal annotation scheme,^17^ models stored in other databases are generally poorly annotated.

Another relevant source of data is the SabioRK database,^11^ which stores kinetic parameters and rate laws for enzymatic reactions. SabioRK tracks provenance of parameter values and supplies the data in SBML format. However while SabioRK stores single enzymatic reactions and can export them in SBML format (in essence, ModelBricks) it does not contain other cellular components such as ion channels or transporters, nor does it contain higher-level ModelBricks that encompass macromolecular assemblies, such as signaling complexes with multiple receptors and kinases.

Lack of annotation makes it non-trivial to reuse existing models or their components when building new models. One rarely reuses an entire existing model, but rather wants to reuse a specific subset of reactions and species from an existing model. Unless components are annotated, there are only limited means to search existing databases for particular reactions or sets of coupled reactions, and it is not possible to directly retrieve only some reactions to add to a new model. Identifying the relevant components within an existing model is also stymied by the fact that a single type of molecule can be modified in multiple ways, making the identification of a molecular species within a model ambiguous.

Realistically, most modelers do not spend the extra time required to fully annotate either model components or the sources of parameter values. Building biological models requires finding quantitative information about all of the components from many different sources including the literature, kinetic reaction databases, pathway databases, and model databases. Each provides pieces of information that need to be combined and therein lies the problem: none of these sources provides the information in modules that could be easily combined. When creating a new model, one typically picks a portion of an existing model (e.g., from BioModels), adds some additional reactions (e.g., from SABIO-RK), and uses a lot of data from the literature for new reactions, parameter values, and initial conditions. The modeler spends significant time sorting through this information to assemble the model structure, but lacks a straightforward way to retain the information sources within the structure of the model. Finally, the provenance of the model itself is also generally not maintained; many existing models are derivative of older models, but sources are rarely referenced (for a good example of the evolution of complex models, see ref. ^23^).

While tools for extracting information from existing models would surely make it easier to build new models, a better approach may be to organize the current models into a collection of well-annotated modules (submodels) that can easily be reutilized in new models. An extremely valuable resource for modelers would be a database that contained these components and their associated properties, already coded in a way that allows them to be readily incorporated into new models. We introduce the term “ModelBricks” to describe such modules.

## Results

### ModelBricks: resuable modules

Assembling models from submodels is not itself a new approach, however a practical design for reuseable, fully annotated and citable components has not been implemented.^24^ A new design is required, having sufficient flexibility to allow modifications and a wide range of scales, while at the same time retaining provenance information and citability. ModelBricks would address this challenge—fully annotated modules that can readily be incorporated into a newly built model, thus allowing models to be built from standard parts.

**Fig. 1:**
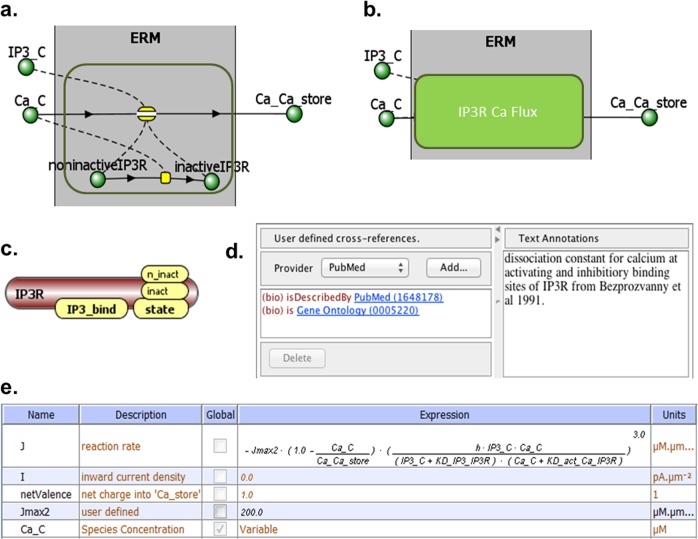
Conceptual ModelBrick for an IP3R calcium channel. **a** A ModelBrick is essentially a reaction mechanism or set of reactions. This hypothetical ModelBrick contains two reactions (yellow) and five species (green); only thfree species are exposed. **b** ModelBrick concept drawing with content hidden. **c** Each species has sites with possible modification states. **d** Prototype annotation panel showing the species “IP3_R” with permanent identifiers. **e** A ModelBrick includes the reaction mechanism and numerical parameters

We envision a ModelBrick to be a small mathematical model that is thoroughly annotated, and assigned a permanent URI that can be referenced. A ModelBrick is a highly structured data object that can encapsulate the current knowledge in the field of a specific type of reaction, small pathway, or process. It would include formal annotation about mechanisms, kinetics, locations relative to other components, and provenance. Thus ModelBricks strive to implement the FAIR principles (Findable, Accessible, Identifiable, and Reusable^25^) and will be invaluable to the broader scientific community beyond their use in creating models. Figure 1 illustrates a theoretical ModelBrick for the IP3R calcium channel used in multiple VCell models from the Hille lab.^26–31^

Essential to creating reusable modular elements for models is the use of standard identifiers for annotation of model elements. It is also essential to be able to identify each species in a model as a composition of uniquely identifiable molecules or moieties, accounting for post-translational modifications and the occupation state of uniquely defined binding sites. A rule-based approach^32,33^ fulfills this requirement, allowing every molecule and its modification state to be linked to databases that contain curated, structured, and queriable information about binding sites and post-translational modifications of biological molecules.

**Fig. 2:**
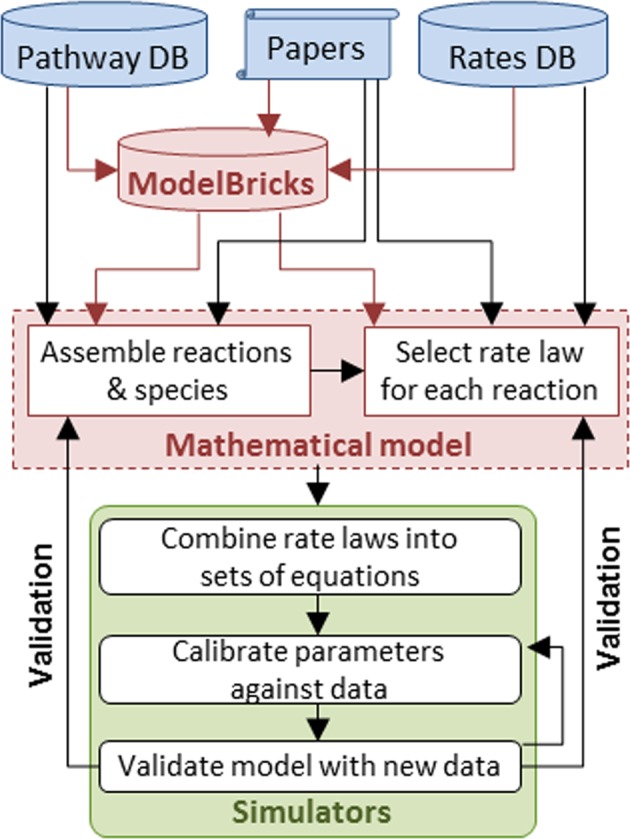
ModelBricks within the modeling workflow. ModelBricks assemble information used to design and annotate a mathematical model. Modeling software creates the math to run simulations the output of simulations using available data sets
- ModelBricks encode common modeling assumptions, thus it is more than a single reaction but less than a complete model.
- ModelBricks encode reaction mechanisms and numerical parameters related to the specific system, so it is more than a part of a pathway.
- ModelBricks simplify model building: elements of published models made available as ModelBricks could be easily incorporated into a new model. For example, the IP3 receptor module (Fig. 1) is reused in at least 6 different models.^26,27,29–31,34^ Furthermore, BioModels database has 35 models that include EGF binding to its receptor, EGFR. Six of those models^35–40^ have exactly the same reactions related to EGF binding: ligand-receptor binding, dimerization and phosphorylation. The parameters are different in these six models, but the kinetics laws are the same; variations of these three reactions are used in about two dozen other models. Currently there is no straightforward way to search for models using the same subset of reactions—a modeler designing a new model of EGFR may know about previous EGFR models in the database, but may need to recode these interactions in a new model, even if it is in SBML. Having ModelBricks of EGF-receptor binding in place, construction of a new model will be easier. Once included the ModelBrick could be modified as needed by the modeler to fit the current modeling context.
- Each ModelBrick will have a permanent URI; this would enable automatic model provenance. Models made of ModelBricks will inherit URIs of all ModelBricks used in the model. Digital Object Identifier (DOI) registration will provide persistent citable references.
- Using ModelBricks within a model would automatically result in at least partially annotated models (as a ModelBrick is fully annotated all model element derived from this ModelBrick are annotated), thus simplifying curation of the complete model.
- ModelBricks would use rule-based modeling features to provide molecular structure for all species where it is applicable. Most reactions, while visible to a modeler as a simple reaction, will be in fact reaction rules that specify a change in molecule modifications or represent binding of molecules through specified binding sites. Individual molecules, their binding sites and posttranslational modifications, and reactions and rules will be linked to stable identifiers.
- Each ModelBrick will have compartment Identifiers that identify a unique cellular compartment using either Systems Biology Ontology (SBO) or Gene Ontology.
- ModelBricks will include numerical parameters specific to a particular application of the reaction or pathway. For example, concentrations included for species of a ModelBrick will assign initial concentrations appropriate to a specific cell type and status. All numerical parameters will be annotated either through references to publications or databases, or by text description.
- ModelBricks will have provenance annotations, recording (a) explicit assumptions in the reaction mechanisms, (b) the granularity of a particular reaction or set of reactions using a new ontology of physical scales and (c) why the component was described with a given mathematics.
- ModelBricks will enable tracking a “family tree” of models showing how modeling of specific pathways evolves.^23^

Key features of the ModelBricks concept:

- ModelBricks are an intermediate data object (Fig. 2) between an excessively large pool of reactions and pathways stored in biological entities databases and a relatively small pool of well-annotated complete models stored in model databases.

### Building models from submodels

Using ModelBricks to build new models will require their assembly and/or integration into the larger model. Enabling assembly of models from submodels has been discussed for some time, with varying degrees of success in implementing standards and methods to accomplish the task.^24,41–43^ COPASI already includes an interface that provides users tools to manually identify and merge the entities of the two source models that represent the same biological entity, resulting in a combined model. VCell also has “smart cut and paste” to copy and paste entire reactions, including their specific kinetics, between models. An extension to SBML Level 3 for Hierarchical Model Composition^44^ provides a language for supporting composition of models into larger ones, and to include “submodels” as parts of larger models. Thus, there already exists a language standard to use in creating a framework that could assemble ModelBricks into a complex model with appropriate computational methods. The key is to ensure that models so created are available to use in many different software packages.

### A ModelBricks infrastructure

The ModelBrick concept requires an infrastructure to make them easily used by the modeling community. Components of this infrastructure would include a repository, tools to create new ModelBricks, and tools to use ModelBricks to build models.

ModelBricks are in essence small SBML models, and as such could be stored in one of the existing model databases, such as BioModels. However, a dedicated repository is likely to be more useful. As we discuss later, a ModelBricks repository would most likely require a level of specialized curation. The repository would also require a versatile API to facilitate interfacing with SBML modeling software. The repository would need dedicated search tools to allow selection of the most appropriate ModelBrick for the modeling situation, and it would also incorporate built-in tools to visualize, create and operate with ModelBricks.

**Fig. 3:**
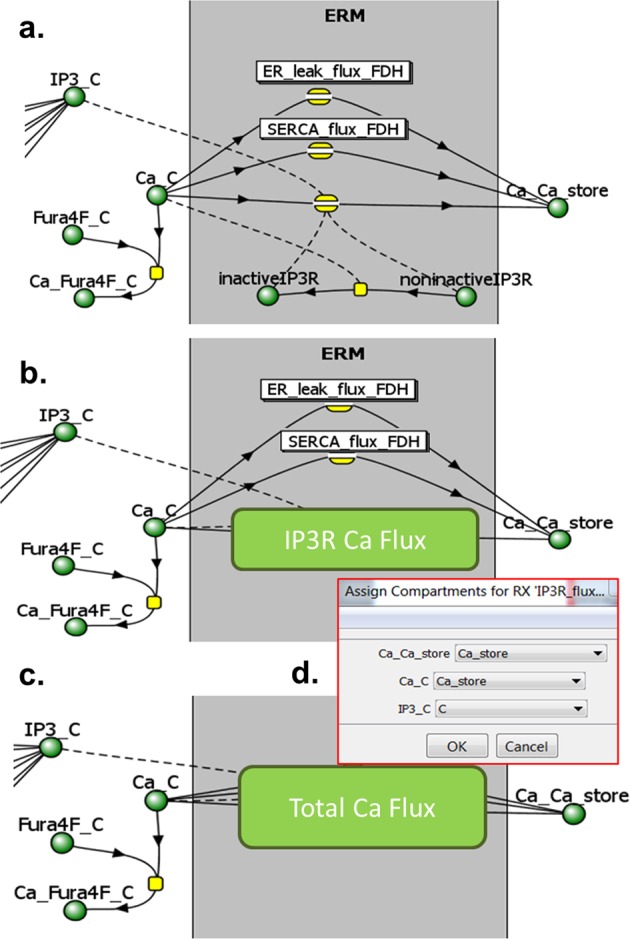
Combining ModelBricks. **a** An initial model with multiple calcium flux mechanisms. **b** The proposed use of an IP3R ModelBrick (Fig. 1) enables better visualization of the diagram but retains the precise context and math. **c** Several mechanisms may be further combined into a new ModelBrick (e.g., Total Ca Flux). **d** A popup window could be used to identify the appropriate compartment for each species when inserting a ModelBrick into an existing model

One can envision multiple ways to create new ModelBricks. A ModelBrick could be created de novo for commonly used reactions or sets of reactions, with parameter values and specified initial conditions, all annotated. ModelBricks can be created with a variety of SBML-compliant tools and be deposited into the repository. A repository user should also be able to create a new ModelBrick either by extracting the components from an existing model, or by modifying an existing ModelBrick for example by changing specific parameters, or (and) by adding additional mechanisms (reactions) to an existing ModelBrick (see for example Fig. 3).

A graphical tool would be useful to visualize a ModelBrick structure (compartments, species, molecules, reactions) and features (kinetic laws, annotations, initial values and parameters), allowing the user to select model elements to remain in the ModelBrick, and delete unnecessary elements. At the same time, new annotations can be added to the ModelBrick. Once done, a ModelBrick can be exported in a variety of formats, such as SBML core, “comp” and “multi” packages, BioPAX, and SBGN.

Modelers may need tools to integrate multiple individual ModelBricks into new, more complex, computable models while preserving the structure of the original ModelBricks. When used within a model, the modeler could change parameter values or initial conditions without losing the ModelBrick structure or provenance. Because a ModelBrick is, in essence, a small SBML model, once added the modeler would also have the ability to “flatten” or remove any ModelBrick structure (but yet retain annotations if the modeling software is compliant). If significant changes to the brick are made in the context of the full model, then it would be possible to remit a new brick back to the repository for use in similar modeling situations. The same reaction or set of reactions can have different mathematical expressions based on different underlying assumptions; it will be possible to have multiple ModelBricks corresponding to different mathematical representations used in peer-reviewed, published models.

Tools for using ModelBricks will need to pay special attention to the organization of compartments in the combined model, suggesting parent-child relationships inherited from GO ontology, but also enabling manual mapping of ModelBrick compartments. Tools for working with ModelBricks will also need hierarchical visualization for ModelBricks, with a compact view hiding and an expanded view presenting all the internal details. Layout and rendering information should be preserved and saved to the SBML ‘layout’^45^ package that describes layout information for models in SBML.

Many issues will require manual intervention when combining ModelBricks, e.g. resolving conflicts between numerical values coming from different ModelBricks that may have different assumptions leading to numerical parameters of different orders of magnitude.

Proposed APIs might include:

- API for searching the ModelBricks database for specific attributes, selecting a ModelBrick and returning it in a chosen format to be added to a model being built using other software.
- API for combining several ModelBricks into a combined model in a chosen format.
- API for uploading a model in supported formats, adding one or more ModelBrick(s), and returning a combined model.

While almost all the ingredients needed for ModelBricks already exist, ModelBricks is still a concept, resembling a synthetic biology concept of designing a new circuit from individual parts, implemented in Synthetic Biology Open Language (SBOL).^46^ The concept resembles the SABIO-RK database that enables export of fully annotated individual reactions as well as merged “models”—SBML files consisting of multiple reactions. However, we envision ModelBricks as more evolved, multi-compartmental models than just a single reaction. ModelBricks could contain, for example, transporters and associated membrane proteins on either side of the membrane. There is a long way to go toward a full implementation of ModelBricks—developing database, tools, and, most importantly, the content. We envision a relational database (MySQL or PostgreSQL) for storing ModelBricks and a Python, command line and REST APIs for invoking plugins for ModelBricks search, retrieval, design and integration into larger models.

## Discussion

ModelBricks would make complex models easier to build, because entire reaction components and mechanisms could be added to a model as self-contained modules. Models constructed of ModelBricks will inherit all of the annotation within the ModelBricks, simplifying annotation of the overall models. We also note that the use of stable identifiers in ModelBricks may benefit database providers themselves, who will be able to track the use of their data in quantitative modeling. This will provide feedback to the database community on the data used in modeling.

ModelBrick URIs could be available prior to publication, and thus be included within PubMed and journal websites at the time of publication. Journals could be encouraged to require properly annotated models at the time of publication. To encourage design and submission of ModelBricks by the community, each could be identified by a DOI with an appropriate citation to the builder, either the citation for a publication including the original model from which the ModelBrick was created, or by citing the ModelBrick database submission. Appropriate citation of a ModelBrick when used will be strongly encouraged. Contributions from the community could be further incentivized by creating a webpage for each ModelBrick, giving full credit to its authors and allowing the ModelBrick to be found in generic web searches.

Resources like the one proposed here only become useful after surpassing a certain critical mass of content. The first question then is how to initiate the ModelBricks repository with a sufficient number of ModelBricks to be useful. The second question is how to engage the community in maintaining it, enabling efficient curation and quality control. The answer to the first question may be surprisingly easy: BioModels is a highly popular source of mathematical models with more than 700 curated models; dissecting these into sets of ModelBricks should be sufficient to create a critical mass of reusable modules. Another easy source for ModelBricks is the SabioRK database, which already provides most of what is needed at the level of single enzyme-catalyzed reactions (though its SBML export would need to be refined to carry annotations). Significant curation effort would be necessary, but much of the work could be carried out in student projects. The second question is more complex. A model built solely from ModelBricks could be sufficiently annotated to constitute a new ModelBrick itself; this could lead to an explosion in the number of ModelBricks. Thus, mechanisms for curation and quality control are necessary for the proposed assignment of DOIs. One model for ongoing curation includes signing by named curators, as is done with the BioModels repository. Another is a peer-review process, incentivized by recognition by the modeling community. A third option is to allow public comments to be associated with a ModelBrick, as it is done in bioRxiv. An extreme option would be to follow the Wikipedia model where editing is done by the community, with changes approved by “editors”. One can also envision an editorial board for a ModelBricks repository, either invited or elected by a ModelBricks community; this model derives from Systems Biology communities working on development of standards, where editors of major community standards such as SBML or BioPAX are elected by the community involved in the development of this particular standard.

There are also issues related to how ModelBricks themselves would be classified and organized. ModelBricks can be created as derivatives of each other, so the provenance of the ModelBricks should be tracked. Once deposited, a ModelBrick must become immutable so it cannot be modified. It should be possible to identify a ModelBrick that contains an error and prevent subsequent usage, creating a new ModelBrick that has been corrected while retaining the original version for provenance. Relationships among ModelBricks will also need to be defined; children and siblings may need special identification.

How to license ModelBricks will have a great effect on their reuse and community development. A Creative Commons Attribution (CC BY) license, used for example in bioRxiv preprints, forces citation; while this might encourage community engagement for design and deposition, it can become a problem when larger ModelBricks are composed of smaller ones, creating a complex web of attributions that can quickly get out of hand. An alternative option that obviates this problem is releasing ModelBricks under CC0, similar to what is done in BioModels. This license allows unrestricted reuse, but does not necessary guarantee a citation.

Multiple levels of quality control are possible, with increasing demands on curation. Completeness of annotation can be verified automatically, requiring each model component to have an appropriate identifier. Checking for annotation consistency can also be automated to some extent, for example flagging for manual correction when the annotation for a process is not consistent with annotations for participating species. Biological consistency can only be curated manually—ensuring that annotations make sense biologically, that parameters are realistic and references lead to the correct sources. Note that when new ModelBricks are derived from existing ones, an automated comparison can be performed so that only new or altered annotations need to be curated. The final level is verification of the mathematics in the reaction kinetics. Since the ModelBrick is used upstream of creating simulation methods and parameters, a true verification of the mathematics cannot be carried out in isolation; rather, the reliance will be that the mathematical form is true to that found in the publication of the model from which the ModelBrick was derived. In the long run, it is up to the modeler to ensure that any model created by assembling ModelBricks is mathematically consistent and all numerical parameters used are properly documented.

While computational cell biology is one arena that will greatly benefit from ModelBricks, it is important to stress that the concept is flexible and can be broadly applied to other types of computational modeling. ModelBricks can include components beyond the cellular scale such as used in physiological, toxicological and pharmacokinetic models. Microbiology and biotechnology models often include cell growth, either by representing a “biomass reaction” (e.g., in genome-scale metabolic models) or by direct cell division; here too ModelBricks would be able to represent “biomass reactions” appropriate in different contexts, and modules for cell division. The latter example extends its usefulness also to studies of the microbiome and ecological models that study the collective metabolism of a microbial community; and indeed also to chemical and environmental engineering models. The ModelBricks concept can provide a concrete method to both enhance reusability of mathematical models and to simplify model construction in a wide range of applications.
